# Chinese Herbal Formulas Miao-Yi-Ai-Tang Inhibits the Proliferation and Migration of Lung Cancer Cells through Targeting *β*-Catenin/AXIN and Presents Synergistic Effect with Cisplatin Suppressing Lung Cancer

**DOI:** 10.1155/2020/2761850

**Published:** 2020-01-16

**Authors:** Bo Li, Wei Zhang, Tao Tan, Wei Liu, Xian Luo, Jun Zhang, Yi Yang, Ruogu Li, Zhengxing Ge

**Affiliations:** ^1^Department of Respiratory Medicine, The First Affiliated Hospital of Guangzhou University of Traditional Chinese Medicine, No. 16, Baiyun Airport Road, Baiyun District, Guangzhou, Guangdong Province, China; ^2^Department of Respiratory Medicine, The Second Affiliated Hospital of Guizhou University of Traditional Chinese Medicine, No. 32, Feishan Road, Yunyan District, Guiyang 550001, Guizhou Province, China

## Abstract

**Objective:**

Lung cancer is one of the major causes of cancer deaths worldwide, and the five-year survival still remains low despite the improvement of screening, prevention, and treatment methods. Chinese herbal medicines have been widely used for tumor prevention and treatment. Miao-Yi-Ai-Tang (Miao) is a novel herbal formulation and shows a potential anticancer effect. *Materials and Methods*. Human Small Cell Lung Cancer Cell was used for study in vitro. After treatments by Miao and Cisplatin (DDP), the invasion, migration, proliferation, and apoptosis of cells were detected by transwell, wound healing, CCK-8, and flow cytometry, respectively. The expression of *β*-catenin, AXIN, and c-myc was detected by qRT-PCR and immunohistochemistry staining. Western blotting was applied for measuring the protein expression of *β*-catenin, AXIN, and c-myc was detected by qRT-PCR and immunohistochemistry staining. Western blotting was applied for measuring the protein expression of

**Results:**

We found that Miao could inhibit invasion, migration, and proliferation and promote apoptosis of human lung cancer cells. Meanwhile, Miao and DDP presented synergy regulating the proliferation and apoptosis of lung cancer cells. The percentage of lung cancer cells in S and G2 stages was increased markedly by Miao. Besides, the expression of c-myc, AXIN, and *β*-catenin, AXIN, and c-myc was detected by qRT-PCR and immunohistochemistry staining. Western blotting was applied for measuring the protein expression of

**Conclusions:**

Chinese herbal formulas Miao could suppress lung cancer through targeting the *β*-catenin/AXIN signaling pathway. Therefore, our findings may provide a novel strategy for the prevention and treatment of lung cancer.*β*-catenin, AXIN, and c-myc was detected by qRT-PCR and immunohistochemistry staining. Western blotting was applied for measuring the protein expression of

## 1. Introduction

Lung cancer is one of the major malignant tumors and characterized by high mortality throughout the world despite great development in diagnosis and treatments [1, 2]. Several reports indicated that the five-year survival of lung cancer is no more than 20% [3, 4]. Cisplatin (DDP) has been commonly used as first-line medicine to treat many tumors including lung cancer [5]. However, the major problem facing lung cancer patients is the resistance to DDP, and the mechanisms underlying the chemotherapy resistance remain unclear. Several therapeutic strategies including Xeroderma pigmentosum [6], CCAT1 [7], and microRNA-10b [8] have been considered to be a regulator of DDP resistance. However, there should be a long way before they could be used in the clinic.

Chinese herbal medicines have acted a key role in preventing and treating diseases in China for a long time. It was reported that QHF (Q, Qingrejiedu; H, Huoxuehuayu; and F, Fuzhengguben) could inhibit the migratory and invasive activity of liver cancer cells [9]. Meanwhile, the Ze-Qi-Tang formula could suppress the growth of non-small-cell lung cancer cells by regulating p53 [10]. Moreover, San Huang Decoction may suppress breast cancer growth and enhance chemosensitivity to anticancer drugs [11]. Miao-Yi-Ai-Tang (Miao) is a novel herbal formulation consisting of six different herbs including *Hedyotis diffusa*, *Solanum lyratum*, *Cephalotaxus fortunei*, Herba Salviae Chinensis, Fructus Akebiae, *Scutellaria barbata*. The role of Miao in affecting lung cancer has not been unfolded.

In this study, we firstly investigate the influence of Miao on the invasion, migration, apoptosis, and proliferation of lung cancer cells. Meanwhile, the improvement of DDP resistance induced by Miao was also observed. Moreover, the expression of some Wnt signaling pathway proteins, c-myc, AXIN, and *β*-catenin, was measured both in vivo and in vitro. Finally, a subcutaneously transplanted tumor model of lung cancer was established to observe the influence of Miao on tumor growth. This study may provide novel insight into the prevention and treatment of lung cancer.

## 2. Methods and Materials

### 2.1. Herbal Extraction

Miao consisted of *Hedyotis diffusa*, *Solanum lyratum*, *Cephalotaxus fortunei*, Herba Salviae Chinensis, Fructus Akebiae, and *Scutellaria barbata*. The ration of these six herbs was 1 : 1:1 : 1:1 : 1. All the herbs used in this study were obtained from the Chinese Herbal Medicine Dispensary of Shanghai East Hospital. The extract of Miao was prepared by decocting the dried prescription of herbs. Briefly, the raw materials of Miao formulation were mixed and crushed into small pieces. Ten-time volumes of water were added with raw components and boiled for 2 h and extracted five times. The aqueous extract was filtered and stored at 4°C before use. The Miao was diluted with the medium before treating cells.

### 2.2. Cell Culture

Human Small Cell Lung Cancer Cell (NCI-H446) (BeNa Culture Collection, China) was used in this study. Cells were cultured in DMEM (Gibco, USA) containing 5% FBS (Gibco, USA) on the condition of 37°C and 5% CO_2_. After 3 passages, cells were incubated with Cisplatin (DDP, 0.6 *µ*g/ml, Keygene Biotech, China) and different dilutions of Miao for 48 h. Then cells were used for different studies.

### 2.3. Cell Proliferation Assay

Cell proliferation was detected by CCK-8 assay (Keygene Biotech, China). Cells (8 × 10^3^) were seeded into 6-well plates and cultured for 24 h. After treatment with DDP and Miao for 48 h, 20 *µ*L CCK-8 reagent was added. After 2 h, the optical density of each well at 450 nm was detected.

### 2.4. Flow Cytometry Analysis

Flow cytometry analysis was conducted as described above [12, 13]. Cells (8 × 10^3^) were seeded into 6-well plates and cultured for 24 h. After culture with DDP and Miao for 48 h, cells (1 × 10^5^/ml) were collected and suspended in 1 mL binding buffer containing 10 *µ*L Annexin V-FITC and 10 *µ*L propidium iodide (PI) (Keygene Biotech, China). After incubation for 10 min in the dark, apoptosis was measured through flow cytometry. The apoptotic rate was scored by quantifying late apoptosis or necrosis cells (Annexin V-FITC+ PI+) and early apoptosis (Annexin V-FITC+ PI−).

### 2.5. Detection of Cell Cycle

Cells (8 × 10^3^) were seeded into 6-well plates and cultured for 24 h. After culture with DDP and Miao for 48 h, cells (1 × 10^5^/ml) were collected and fixed by 75% alcohol for 2 h. After washing 3 times by PBS, cells were stained by 100 *µ*L PI at 4°C for 30 min. Then, the cell cycle was measured by flow cytometry.

### 2.6. RNA Isolation and Real-Time PCR

RNA isolation and real-time PCR were performed as described previously [14]. Total RNA was extracted from cells using TRIzol reagent (Invitrogen, Carlsbad, CA, USA) and 200 ng RNA was reverse-transcribed into cDNA via SuperScript™ II Reverse Transcriptase (Invitrogen, Carlsbad, CA, USA). cDNA from each group was determined by RT-qPCR using SYBR Premix Ex Taq™ (Takara, Beijing, China). The primer information was listed as follows: (1) *β*-catenin: forward: 5′-AGCCGACACCAAGAAGC-3′ and reverse: 5′-GCACGAACAAGCAACTGAAC-3′; (2) AXIN: forward: 5′-AAGATGGGATAAGCCTGTTC-3′ and reverse: 5′-TCAGCCTCTTCTCCTCGTTC-3′; (3) c-myc: forward: 5′-GAGGAGGAACGAGCTAAAAC-3′ and reverse: 5′-TGCTTGGACGGACAGGATG-3′. Data were analyzed by comparing cycle threshold values. The relative expression of target genes was analyzed using the 2^−∆∆Ct^ method. The fold change between the experimental group and the control group = 2^−∆∆Ct^.

### 2.7. Western Blot Analysis

Western blot analysis was conducted as described previously [15]. Briefly, cells were lysed in a lysis buffer solution. *β*-Catenin in the nuclear was obtained with a nuclear and cytoplasmic protein extraction kit (Beyotime Institute of Biotechnology; Shanghai, China). Protein concentration was measured with the BCA assay (Keygene Biotech, China). 30 ng protein of each sample was purified by 10% SDS-PAGE gels and followed by transfection to a polyvinylidene difluoride membrane. The membranes were blocked with nonfat milk and then incubated with primary antibodies (1 : 1000) at 4°C overnight. After washing and incubation, the membranes were incubated with a secondary antibody (1 : 1500) for 1-2 h in TBST. Quantity One was used to analyze the protein grey.

### 2.8. Transwell Assay

Cell activity was measured through polycarbonate membrane Boyden chambers in a transwell apparatus (Costar, USA). Cells (10^5^) were seeded to the top chamber of the transwell. The lower chamber was added with 2 mL DMEM containing 5% FBS. After incubation (48 h), the lower chamber was fixed with 4% polyformaldehyde (20 min). After washing twice, the lower chamber was stained with Giemsa for 10 min. The number of migrated cells in the five fields was counted using an inverted microscope (Olympus CKX31, Japan).

### 2.9. Wound-Healing Assay

Cells were firstly plated in a 6-well plate and cultured on the condition of 37°C and 5% CO_2_. When the cells reach 70% confluence, a wound was made along the center of every well by a 1 mL pipette tip. After 48 h incubation, images were captured with an inverted microscope (Olympus CKX31, Japan). Three fields for each group were chosen, and the migrated distance of cells was measured by ImageJ software.

### 2.10. Establishment of Subcutaneously Transplanted Tumor Model

Nude mice (C57BL/6, 6 weeks) were purchased from Beijing Vital River Laboratory Animal Technology (Beijing, China). The animal experiments were approved by the Second Affiliated Hospital of Guizhou University of Traditional Chinese Medicine. NCI-H446 cells (5 × 10^6^, 0.1 mL) were subcutaneously injected into the armpits of mice. When the tumor grew to around 8 mm, mice were randomly divided into 3 groups, control, Miao (20 times dilution), and Miao (20 times dilution) + DDP, and each group included 5 mice. Mice were subcutaneously injected with normal saline (0.2 mL), Miao (20 times dilution, 0.2 mL), and Miao (20 times dilution, 0.1 mL) + DDP (20 mg/kg, 0.1 mL), respectively, 1 time twice days. After 21 days, mice were sacrificed and tumors were collected. The weight of the tumor was measured.

### 2.11. Immunohistochemistry Staining

Then tumor tissues were fixed in 10% formalin for 48 h. Then tissues were embedded in OCT compound (Keygene Biotech, China). The embedded tissues were cross-sectioned in 12 *μ*m thickness. After antigen repair, tissues were washed 3 times (3 min/time). 3% H_2_O_2_ was used to culture tissues for 10 min at room temperature. After washing 3 times (3 min/time), 10% goat serum was used for blocking. Then primary antibodies were used to culture tissues at 4°C overnight. After washing, the tissues were incubated with a secondary antibody for 1 h. Then DAB regent was used to incubate tissues, and an inverted microscope (Olympus CKX31, Japan) was used for capturing.

### 2.12. Statistical Analysis

At least three independent experiments were conducted. Data are presented as the mean ± standard deviation (SD). Statistical analysis was calculated using SPSS 20.0. Student's *t*-test was utilized to assess the statistical significance of the difference between two independent groups. *P* values <0.05 were believed to be significant difference.

## 3. Results

### 3.1. Miao Inhibited Proliferation and Promoted Apoptosis of Human Lung Cancer Cells

**Figure 1:**
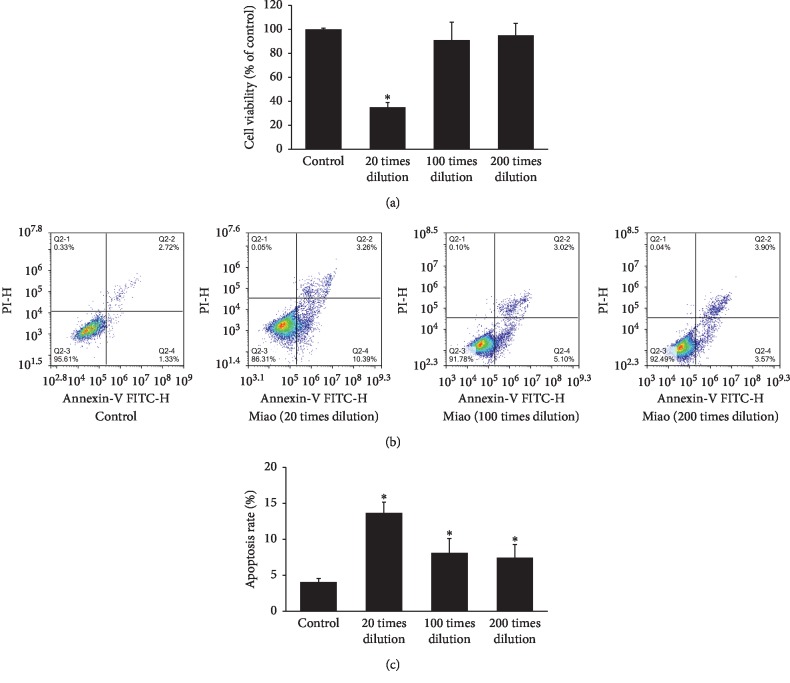
Miao inhibited proliferation and promoted apoptosis of human lung cancer cells. (a) Cell proliferation was measured by CCK-8 assay after treatment with different concentrations of Miao; (b) cell apoptosis was measured after treatment with different concentrations of Miao; (c) quantification analysis of cell apoptosis after treatment with different concentrations of Miao. ^*∗*^*P* < 0.05 compared with the control group.

Firstly, we investigated the influence of Miao with different dilutions on cell proliferation. We found that 20 times dilution of Miao presented a significant suppressive effect on cell proliferation of human lung cancer cells (NCI-H446) (Figure 1(a)). Meanwhile, 20 times dilution of Miao remarkably promoted the cell apoptosis of NCI-H446, and the promoting effect was dose-dependent (Figures 1(b) and 1(c)). Therefore, Miao might play an important role in regulating human lung cancer cells.

### 3.2. Miao and DDP Presented Synergy Regulating Proliferation and Apoptosis of Human Lung Cancer Cells

**Figure 2:**
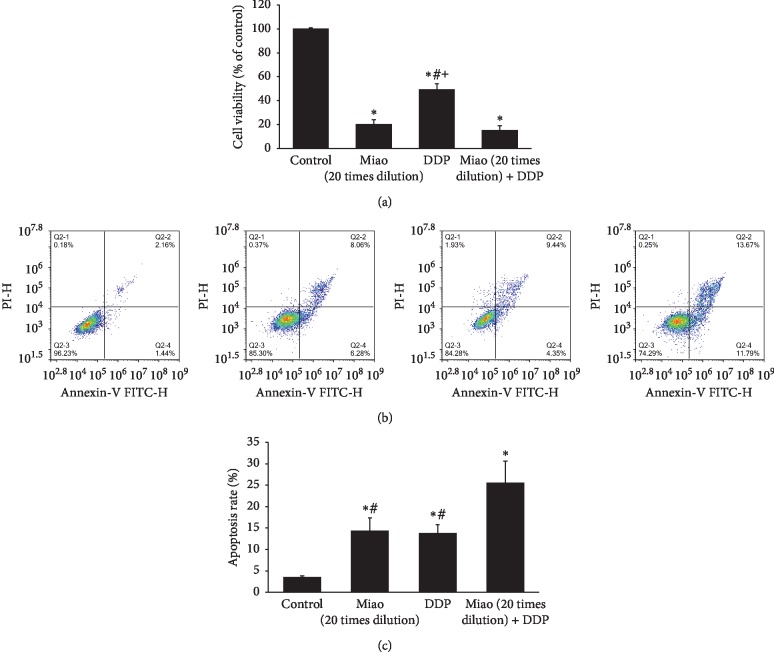
Miao and DDP presented synergy regulating proliferation and apoptosis of human lung cancer cells. (a) Cell proliferation was measured by CCK-8 assay after treatment with Miao and DDP; (b) cell apoptosis was measured after treatment with Miao and DDP; (c) quantification analysis of cell apoptosis after treatment with Miao and DDP. ^*∗*^*P* < 0.05 compared with the control group. ^#^*P* < 0.05 compared with group Miao (20 times dilution) + DDP. ^+^*P* < 0.05 compared with Miao (20 times dilution) group.

DDP is the standard first-line chemotherapeutic drug for lung cancer and has been commonly used for lung cancer patients. In this study, 20 times dilution of Miao and DDP showed a similar influence on cell proliferation and apoptosis of lung cancer cells (Figure 2). Besides, 20 times dilution of Miao presented a higher suppressive effect on lung cancer cell proliferation compared with DDP (Figure 2(a)). Meanwhile, treatment with 20 times dilution of Miao and DDP simultaneously markedly inhibited proliferation and promoted apoptosis of lung cancer cells compared with DDP indicating that Miao and DDP might play a synergy mediating proliferation and apoptosis of lung cancer cells.

### 3.3. Miao Markedly Increased the Percentage of Lung Cancer Cells in G2 and S Stages of the Cell Cycle

**Figure 3:**
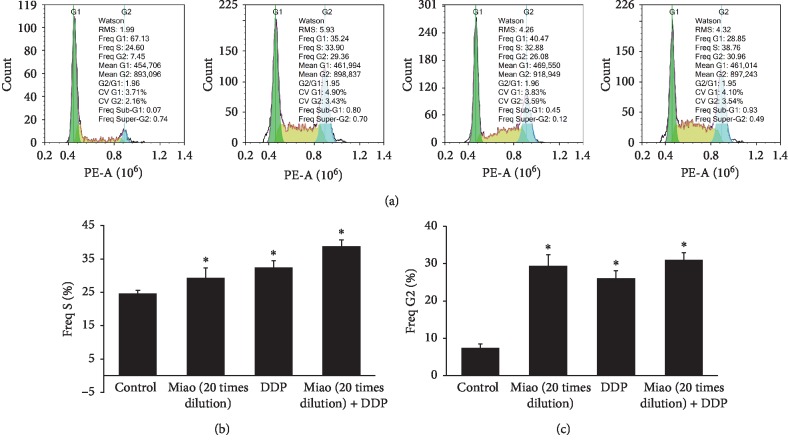
Miao markedly increased the percentage of lung cancer cells in G2 and S stages of the cell cycle. (a) Representative pictures of the cell cycle after treatment with Miao and DDP; (b) quantification analysis of cells in S stage after treatment with Miao and DDP; (c) quantification analysis of cells in G2 stage after treatment with Miao and DDP. ^*∗*^*P* < 0.05 compared with the control group.

To unfold how Miao and DDP influence the death and apoptosis of lung cancer cells, we measured the cell cycle after different treatments. We found that Miao and DDP could markedly increase the percentage of cancer cells in the G2 and S stages of the cell cycle (Figure 3). Meanwhile, the difference in the percentage of cells in the G2 and S stages between 20 times dilution of Miao and DDP was not significant (Figure 3).

### 3.4. Miao Markedly Inhibited the Migration and Invasion of Lung Cancer Cells

**Figure 4:**
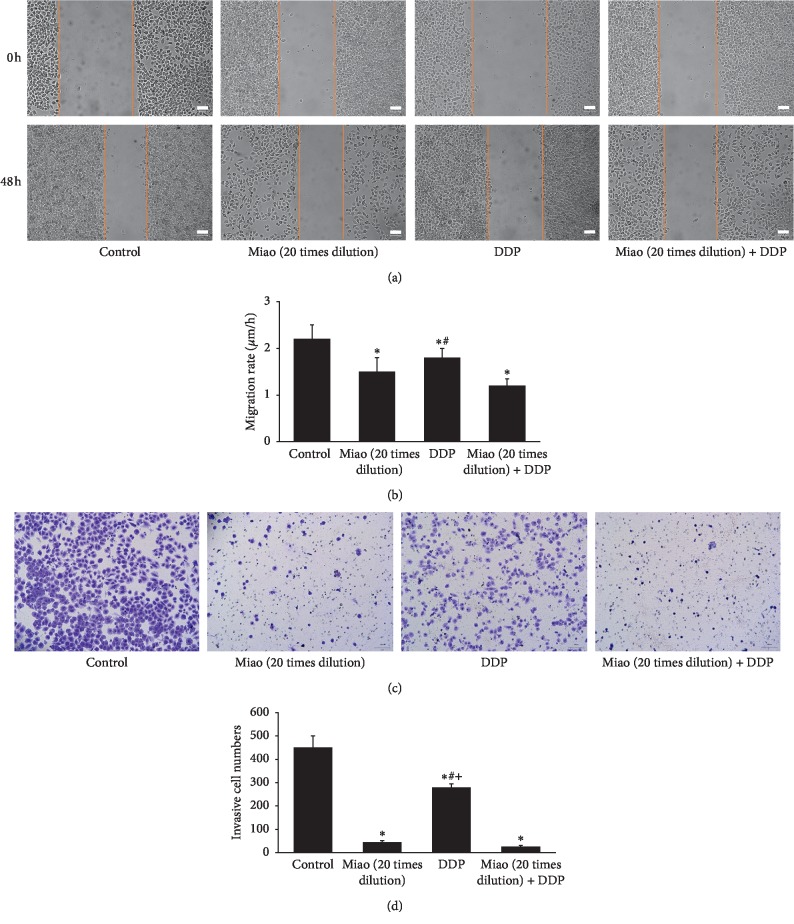
Miao markedly inhibited the migration and invasion of lung cancer cells. (a) Representative pictures of cell migration after treatment with Miao and DDP; (b) quantification analysis of cell migration after treatment with Miao and DDP; (c) representative pictures of cell invasion after treatment with Miao and DDP; (d) quantification analysis of cell invasion after treatment with Miao and DDP. ^*∗*^*P* < 0.05 compared with the control group. ^#^*P* < 0.05 compared with group Miao (20 times dilution) + DDP.

Cell migration and invasion have been believed to be closely related to tumor metastasis. We investigated the influence of Miao and DDP on the migration and invasion of NCI-H446. We found that both Miao and DDP could significantly inhibit the invasion (Figures 4(c) and 4(d)) and migration (Figures 4(a) and 4(b)) of lung cancer cell, and treatment with 20 times dilution of Miao and DDP simultaneously presented even stronger suppressive effect compared with DDP treatment only (Figures 4(b) and 4(d)). Additionally, the inhibiting effect on cell invasion caused by Miao was significantly higher than DDP (Figure 4(d)). Therefore, Miao may enhance the anticancer effect of DDP.

### 3.5. Miao Markedly Inhibited the Expression of c-myc, AXIN, and *β*-Catenin

**Figure 5:**
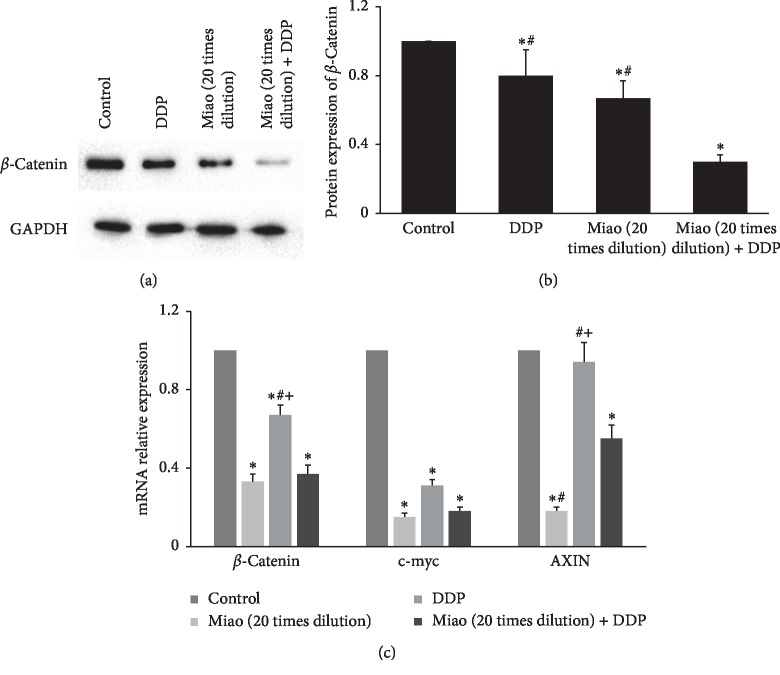
Miao markedly inhibited the expression of c-myc, AXIN, and *β*-catenin. (a) Western analysis of *β*-catenin after treatment with Miao and DDP; (b) protein expression of *β*-catenin after treatment with Miao and DDP; (c) mRNA expression of c-myc, AXIN, and *β*-catenin after treatment with Miao and DDP. ^*∗*^*P* < 0.05 compared with the control group. ^#^*P* < 0.05 compared with group Miao (20 times dilution) + DDP. ^+^*P* < 0.05 compared with Miao (20 times dilution) group.

c-myc, AXIN, and *β*-catenin are considered as the major targets of the Wnt signaling pathway and their abnormal expressions have been believed to be closely linked with the progression of cancers. Therefore, we investigate the influence of Miao and DDP on their expressions. We found that both DDP and 20 times dilution of Miao could remarkably inhibit the protein and mRNA expression of *β*-catenin, and the treatment with Miao and DDP simultaneously presented an even stronger suppressive effect (Figure 5). Meanwhile, the similar influence of Miao and DDP on mRNA expression of c-myc was also observed (Figure 5(c)). Interestingly, no significant difference was observed between DDP and the control group on the mRNA expression of AXIN (Figure 5(c)), but Miao dramatically inhibited the mRNA level of AXIN (Figure 5(c)).

### 3.6. Miao Remarkably Suppressed Tumor Growth and the Expression of c-myc, AXIN, and *β*-Catenin In Vivo

**Figure 6:**
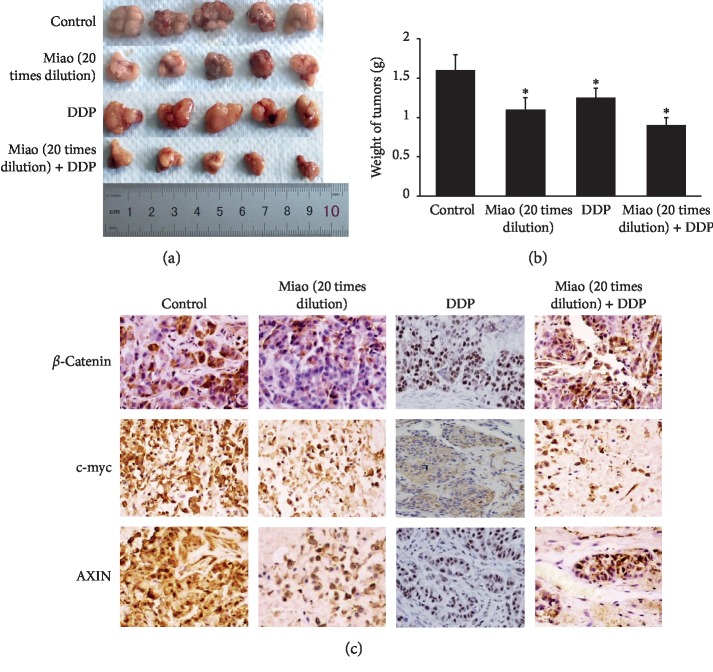
Miao markedly inhibited the growth of tumor and expression of c-myc, AXIN, and *β*-catenin in vivo. (a) Representative pictures of tumor after different treatments; (b) measurement of tumor weight; (c) expression of c-myc, AXIN, and *β*-catenin was measured by immunohistochemistry staining after treatment with Miao and DDP. ^*∗*^*P* < 0.05 compared with the control group.

To further investigate the influence of Miao and DDP on lung cancer in vivo. We established the transplanted tumor model models and treated nude mice with Miao and Miao + DPP. We found that both Miao and Miao + DPP presented a significant suppressive effect on tumor growth in vivo (Figures 6(a) and 6(b)). No significant difference between group Miao and group Miao + DDP was observed (Figure 6(b)). Meanwhile, the expression of c-myc, AXIN, and *β*-catenin in tumor tissues was also investigated by immunohistochemistry staining, and the results were in line with previous findings in vitro. The expression of c-myc, AXIN, and *β*-catenin in tumor tissues was markedly inhibited by treatment with Miao or Miao + DDP (Figure 6(c)). These results in vivo further confirm the suppressive role of Miao on lung cancer.

## 4. Discussion

Because of the abuse of tobacco, pollution of the environment, and aging of populations, the incidence of lung cancer is increasing significantly in the past decades [16, 17]. Moreover, despite the improvement of chemotherapy, radiotherapy, and surgical treatments, the five-year survival of lung cancer remains remarkably lower than most other types of cancers [18, 19]. DDP has been widely used as a primary therapeutic strategy against malignancies including lung, ovarian, esophageal, and gastric organs [20, 21]. However, DDP resistance has been observed during the treatment of tumors, which affects the therapeutic effect [22, 23]. Therefore, it is necessary to explore new types of drugs for the treatment of lung cancer.

Chinese herbal medicines play a crucial role during several thousands of years, and due to the relatively low toxicity and effective treatment, they are still welcomed by many patients from China and other countries. Miao is a type of traditional Chinese herbal medicine, and it consists of six different kinds of herbs. We found that Miao could suppress the proliferation, migration, and invasion of lung cancer cells (Figures 1 and 4). Meanwhile, the apoptosis of cells was promoted remarkably (Figure 1(c)). Additionally, we also measured the effect of treatment with Miao and DDP simultaneously on lung cancer cells. Interestingly, treatment with Miao and DDP simultaneously could markedly strengthen the influence of DDP on lung cancer cells. Additionally, both treatment with Miao and Miao + DDP could remarkably inhibit the tumor growth in vivo (Figures 6(a) and 6(b)). Therefore, Miao might be a promising medicine to inhibit lung cancer and reduce DDP resistance. Both Miao and DDP significantly increased the percentage of cells in S and G2 stage suggesting that they may possess similar functioning mechanisms targeting lung cancer cells.

Wnt/*β*-catenin pathway has been proved to be closely related to the progression and development of cancers [24–26]. *β*-Catenin is a key factor in the Wnt/*β*-catenin signaling pathway, and the activation of Wnt signaling could lead to the accumulation of *β*-catenin in the cytoplasm, which promotes the transcription of c-myc [27]. As the cytoplasmic anchor of *β*-catenin, AXIN could facilitate the accessibility of *β*-catenin to the cell nucleus and thereby activate downstream targets [28]. Therefore, we measured the influence of Miao and DDP on the expression of *β*-catenin, AXIN, and c-myc both in vivo and in vitro. The inhibition of *β*-catenin, AXIN, and c-myc by Miao and DDP indicates that Miao and DDP might regulate the proliferation, migration, invasion, and apoptosis of lung cancer cells through targeting the Wnt/*β*-catenin pathway.

In summary, we demonstrated that Miao and DDP presented a similar influence on lung cancer cells, and treatment with Miao and DDP simultaneously could strengthen the suppression effect on lung cancer cells. This study may provide a new thought for the treatment of lung cancer.

## Data Availability

The data used to support the findings of this study are available from the corresponding author upon request.

## Abbreviations

Miao: Miao-Yi-Ai-Tang
DDP: Cisplatin
QHF: Q, Qingrejiedu; H, Huoxuehuayu; and F, Fuzhengguben
SD: Standard deviation
